# Cross-attention guided discriminative feature selection for robust point cloud domain generalization

**DOI:** 10.1371/journal.pone.0329146

**Published:** 2025-08-13

**Authors:** Jiajia Lu, Wun-She Yap, Kok-Chin Khor

**Affiliations:** 1 Fuzhou Institute of Technology, School of Electronic Engineering, Fuzhou, Fujian, China; 2 Universiti Tunku Abdul Rahman, Lee Kong Chian Faculty of Engineering and Science, Kajang, Selangor, Malaysia; Kafkas University: Kafkas Universitesi, TÜRKIYE

## Abstract

In recent years, deep learning networks have been widely employed for point cloud classification. However, discrepancies between training and testing scenarios often result in erroneous predictions. Domain generalization (DG) aims to achieve high classification accuracy in unseen scenarios without requiring additional training. Although current DG methodologies effectively employ data augmentation and representation learning, they inadvertently neglect a key component: discriminative feature selection, which we identify as a crucial missing element for achieving robust domain generalization. To fully leverage the geometric features of point clouds, we propose a novel domain generalization method that emphasizes transferring contextual information to improve generalization performance for 3D point clouds. Our method projects the point cloud into multiple views and employs a 2D adaptive feature extractor to capture and aggregate weighted semantic features, while leveraging the DGCNN network to extract 3D spatial geometric features. Additionally, we incorporate an attention mechanism to fuse 2D semantic features with 3D geometric features, facilitating the selection of discriminative features from point clouds. The experiments demonstrate that our method outperforms state-of-the-art methods in both multi-source and single-source tasks, achieving superior generalization performance.

## Introduction

Point clouds have become a popular 3D data representation due to their rich spatial geometric information and ease of acquisition from LiDAR devices [1]. Point cloud object classification has recently emerged as a hot research area, with significant applications in autonomous driving, virtual reality, and robotics. Developing deep learning networks for point clouds has effectively tackled classification and object detection challenges. These deep learning networks for point clouds are trained using labeled data to make predictions. To mitigate the high cost of annotation, scholars have explored leveraging labeled source domains to predict unlabeled target domains, a technique known as domain adaptation (DA) [2, 3]. However, DA addresses the challenge of predicting objects only in specific target domains. In many scenarios, the target domain is inaccessible and cannot be incorporated into the training process. The emergence of domain generalization (DG) offers a solution to this limitation [4]. DG is an approach that focuses on leveraging source domains to learn generalized features and make predictions on unseen target domains. It is widely applied across various fields [5]. In autonomous driving, DG enables the recognition of scenarios in unseen cities, while in medical image analysis, it helps in interpreting images from diverse or uncertain equipment. Remarkably, DG can help to recognize the images which has an obvious distinction from the training dataset and make the minimum prediction error in classification [4].

Domain adaptation (DA) addresses the task of predicting known target domains by aligning features between the source and target domains. Meanwhile, domain generalization (DG) presents a greater challenge, as it aims to extend the model’s generalization capabilities using only source domain data, without access to the target domain during training. The main methods for implementing DG include data augmentation, representation learning, and learning strategy [4]. Data augmentation is widely used to improve generalization performance and includes methods such as expanding the dataset through image style transfer [6], masking partial structures [1], adversarial data generation [7], among others. These methods primarily serve to expand the scale and diversity of training data. Xiao *et al*. [1] propose an adversarial strategy in the multi-source domain to explore domain-invariant features through representation learning. Huang *et al*. [8] employs meta-learning to learn point cloud representations from a set of classification tasks on transformed point sets. These proposals primarily focus on leveraging source domain augmentation or inter-domain interactions in multi-source domains, often failing to identify and preserve the most discriminative and generalizable features within the source domain. To bridge this gap, we propose a novel approach that enhances generalization by more effectively identifying and leveraging discriminative intra-domain features.

**Fig 1:**
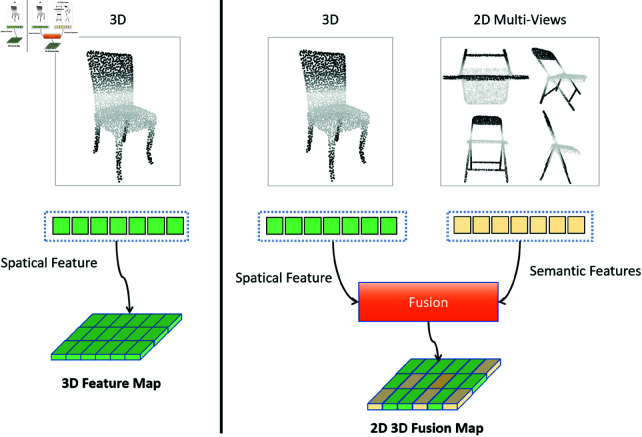
We employ multi-view projections derived from raw point cloud data. The multi-view features are concatenated to extract semantic features, while the raw 3D data is processed to capture spatial features. These global features subsequently guide the fusion of multi-view image features and 3D neighborhood features through a cross-attention mechanism.

3D point clouds provide precise spatial structures, while their corresponding 2D images offer rich semantic information. Traditional point cloud processing methods typically handle 3D point cloud data independently, failing to fully exploit the geometric discriminative features of point clouds. Xu *et al*. [9] proposed PointFusion, which achieves feature-level fusion between 3D point clouds and 2D images. This multi-modal approach allows complementary data to compensate for individual limitations, thereby improving classification robustness and accuracy. However, since these modalities are usually captured by separate sensors (LiDAR and RGB cameras), they require complex cross-sensor calibration. In contrast, our method extracts both 2D and 3D features directly from single-sensor input, eliminating calibration requirements while maintaining fusion performance. As shown in Fig 1, multi-view feature extractors have been observed to provide rich contextual semantic information [10, 11]. Furthermore, 3D neighborhood-based features effectively capture spatial position information. In this paper, we leverage the point cloud geometry information and employ the attention mechanisms to fuse the 2D and 3D features to obtain the discriminative representation for improving the model’s generalization. Our contributions are summarized as follows:

(1) We project the raw point cloud into multiple views and propose an adaptive feature extraction to encode 2D multi-view features.

(2) We utilize a cross-attention module to select the relevant transferable information between the 2D and 3D features.

(3) We evaluate our proposed method on the PointDA-10 dataset. The experimental results show that the average accuracy improves by 1.2% in multi-source mode and 2.6% in single-source mode compared to the state-of-the-art method.

## Related work

### Point clouds representation learning

**Fig 2:**
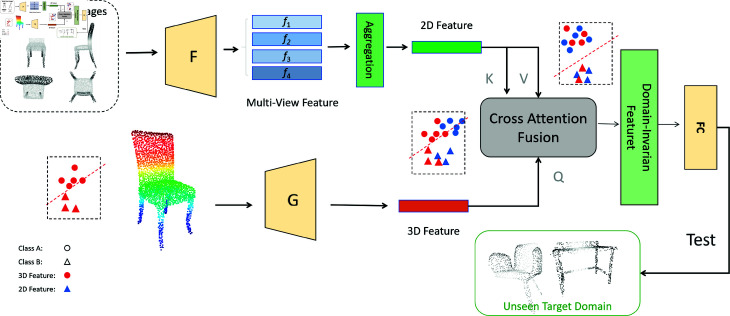
Firstly, DGCNN leverages the direct relationships between points and their neighborhoods through edge convolution, effectively capturing global spatial features. Concurrently, the original point cloud data is projected into multiple views, where a 2D adaptive feature extractor is applied to obtain semantic features. To integrate these complementary features, a cross-attention module is employed, enabling the fusion of 3D spatial and 2D semantic representations into more discriminative features. Finally, the model is evaluated on unseen target domains to assess its accuracy and generalization performance.

3D shapes can be represented in various forms, including depth images, multi-view images, voxels, point clouds, meshes, and implicit surfaces, among others [11]. The performance of applications is highly dependent on the selected representation [10]. The spatial neighborhood representation can better describe the global information of point cloud objects, and image representations can provide more stable geometric feature information. Point cloud samples in real scenes are often incomplete due to missing parts and occlusions, images captured from different angles offer complementary information for inferring 3D objects [10]. It has been observed that multi-view representation more effectively captures stable and rich contextual semantic features. Fig 2 shows the visualization of the ScanNet dataset from ten different views.

The point cloud is a set of unordered points scanned from a 3D surface, with each point described by its position in three-dimensional space [10, 12]. DGCNN develops the geometric structures of point clouds by constructing the local adjacency graph and applying edge convolution to extract point cloud features [13], and it has been proven to obtain global feature information of point clouds effectively. ResNet [14], MobileNet [15], and other deep learning convolutional networks are implemented to encode the two-dimensional projection images. ResNet-50 incorporates residual connections to mitigate gradient vanishing or explosion, enabling deeper and more stable network training. This architecture exhibits strong generalization capabilities, demonstrating consistent adaptability across diverse datasets and tasks. Notably, it achieves robust performance in various imaging domains, including medical image analysis and 2D projections of point cloud data [16]. However, the challenge of the multi-view representation is effectively integrating the features from each view [17]. MVCNN [18] proposes using a standard CNN to train the rendered views of shapes independently and then combines information from multiple views into a single compact shape descriptor. PointCLIP [12] projects the raw point cloud data into multiple views and encodes each view’s information into fixed features, then concatenates the independent multi-view features to obtain a global representation through two Multi-Layer Perceptrons (MLPs). PointMCD [19] explores the multi-view cross-modal distillation from a 2D image encoder, acting as the teacher, to a 3D point deep learning encoder, acting as the student. BEV-DG [20] began applying cross-modal learning in domain generalization, capturing both 2D and 3D features and pushing the 2D and 3D networks to learn domain-invariant features jointly.

### Domain generalization

Domain generalization (DG) has been receiving increasing attention as it effectively addresses cross-domain problems. While significant progress has been made in DG for 2D image tasks, its application to 3D point cloud data remains limit [21]. The main domain generalization methods are categorized into three types: data manipulation, representation learning, and learning strategy [4, 22]. Data augmentation has become a standard paradigm for domain generalization due to its proven effectiveness in enhancing generalization. Volpi *et al*. proposed to employ an adversarial strategy to generate the difficult sample that augments the dataset [7]. Cugu *et al*. [23] use the visual corruptions as an augmentation to achieve the single source domain generalization. Data augmentation is also applied to 3D point clouds through techniques such as rotation, scaling, shearing, flipping, and translation with point-wise jittering [23]. Xu *et al*. [6] proposed designing a learnable 3D data augmenter to generate new training point clouds by push and pull strategy.

Representation learning is also an important method for domain generalization, the goal of representation learning is to minimize the discrepancies across domains by learning the domain-invariant feature [4]. In the DA field, the discrepancy is reduced by aligning the features of the source and target domains. However, in DG, since the target domain is unseen, discrepancy reduction can only be achieved within the source domain. Huang *et al*. [21] designed a sub-domain alignment method called SUG that forces the learned representations to be more discriminative through the feature alignment between the sub-domains from the single source dataset, Hang *et al*. [1] began to explore the effect of multi-source domains for point clouds DG, it employs a multi-source domain feature alignment by maximum mean discrepancy(MMD) measure to minimize the distribution difference between two source domains and learn a more general representation. The above methods primarily focus on using data augmentation to enhance the model’s generalization performance. Alternatively, some approaches leverage multi-domain learning to extract domain-invariant features. However, less attention has been given to the importance of selecting geometrically relevant domain-invariant features for achieving effective point cloud generalization.

### Attention mechanism

The attention mechanism dynamically focuses on different parts of the input, effectively extracting key information relevant to the current task. Capturing spatial dependencies between local and global points is especially important for recognizing complex geometric structures. Several approaches utilize attention mechanisms to extract discriminative features, thereby enhancing the accuracy of point cloud classification, such as [24–28]. Gao *et al*. propose a 3D point cloud classification method based on a self-attention mechanism that learns robust latent representation [24]. Wang *et al*. [29] leverage the cross-attention and self-attention mechanisms to design a neural network for point cloud completion with implicit local region partition. Yue *et al*. [30] propose a 3D point cloud classification method based on global attention and adaptive graph convolution. Given its ability to capture consistent contextual information across different domains, the attention mechanism is well-suited for application in the domain adaptation (DA) field. Wang *et al*. [31] captured global domain information using the self-attention module and aligned the source and target domains by extracting identifiable features through the dual domain channel branching. Due to its ability to extract more discriminative features, attention mechanisms significantly enhance domain generalization.

Meng *et al*. [32] employed intra-model and inter-model attention diversification regularization for DG. Zhao *et al*. [33] proposed a dual-attention discriminative DG framework, employing multi-scale self-attention to extract spatial features and multi-head external attention to extract spectral features for hyperspectral image classification. Although these methods successfully leverage attention mechanisms to enhance feature discriminability, their exploration of point cloud DG remains limited, as they do not specifically account for the geometric irregularities inherent in point cloud data.

## Methodology

### Problem statement

In typical DG problem settings, given labeled dual source samples $S=\{(P_{i},L_{i}){\}}_{i=1}^{n_{s}}$ and unseen target samples $T=\{(P_{i},L_{i}){\}}_{i=1}^{n_{t}}$, *P* is defined as the sample of point cloud data, and *L* is defined as the label of point cloud data. *n* represents the number of samples. Both *P* and *L* in the source domain are accessible, while *P* and *L* in the target domain are inaccessible. We intend to train a model using the source domain and test it on the target domain to achieve better accuracy.

### Overview

As shown in Fig 2, firstly, our method projects the original point cloud data into multiple views to generate feature vectors for each projection, which are then integrated to form multi-view features. In a separate branch, we employ the DGCNN to extract raw point cloud features. A cross-attention mechanism is then utilized to fuse the 2D and 3D features, enhancing the discriminative ability of the domain features. Finally, we evaluate the trained model on unseen target domains. We discuss depth map projection, feature extraction, and fusion in the following subsections.

$$f_{3d}=\theta (S),f_{2d}=\phi (S)$$ (1)

$$f=Attention(f_{3d},f_{2d})$$ (2)

### Depth map projection

Each point in the point cloud data is represented by a three-dimensional coordinate P=(X,Y,Z). To generate a depth map, it is necessary to project the point cloud onto a two-dimensional plane [34]. This projection is accomplished by mapping each 3D point within the point cloud onto a 2D image plane, concurrently documenting the depth value associated with each pixel [35]. The generation of depth maps from point clouds encompasses several sequential steps: transformation of the coordinate system, projection of points from the camera coordinate system onto the image plane, allocation of depth values into image coordinates, and ultimately, the creation of the depth map utilizing the point cloud data. As illustrated in Eq 3, a point *P* in the world coordinate system is transformed into the corresponding point $P^{\prime }=(X^{\prime },Y^{\prime },Z^{\prime })$ in the camera coordinate system through a rigid transformation.

$$P^{\prime }=RP+T$$ (3)

where *R* represents the rotation matrix of the camera, and *T* denotes the translation vector. This transformation enables the conversion of point clouds from the world coordinate system to the camera coordinate system by applying the respective rotation and translation operations. Eq 4 defines the mapping from 3D camera coordinates to 2D image coordinates, which is essential for projecting 3D points onto the image plane. The corresponding pixel coordinates (*u*,*v*) in the image plane are computed as follows:

$$u=\frac{X^{\prime }}{Z^{\prime }}\cdot \frac{W}{H}\;v=\frac{Y^{\prime }}{Z^{\prime }}$$ (4)

where $\left(X^{\prime },Y^{\prime },Z^{\prime }\right)$ are the coordinates of a point in the camera coordinate system, *W* and *H* represent the width and height of the image, respectively. Eq 5 denotes a transformation from normalized image coordinates to pixel coordinates as follows:

$$u^{\prime }=\frac{(u+1)\cdot W}{2}\;v^{\prime }=\frac{(v+1)\cdot H}{2}$$ (5)

This transformation is essential for converting points from a normalized coordinate system to the actual pixel coordinates of an image. It ensures that the coordinates are scaled and shifted appropriately to fit the image dimensions. Each 3D point is mapped to the image coordinate system through a specific projection method, and the depth map is subsequently generated based on the weighted average of the depth values at each pixel.

### Feature extractor

To comprehensively extract features from point clouds, we leverage both their 2D and 3D representations. In the 2D encoder branch, the point cloud is projected into multiple views, and ResNet-50 is utilized to extract 2D semantic features. In the 3D encoder branch, DGCNN is employed to extract 3D features by dynamically constructing graph structures from the point cloud and its neighboring points.

**Fig 3:**
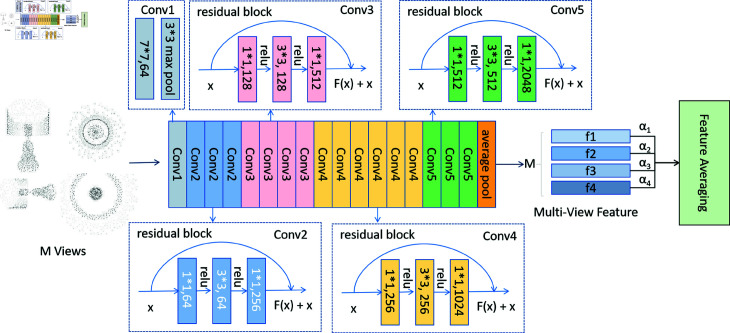
We project the point cloud into multiple views, resizing each view into a 224×224×3 image. These images are then fed into the ResNet-50 network to extract deep semantic features. ResNet-50 consists of an initial convolutional layer, 48 residual blocks, and an average pooling layer. The initial convolutional layer consists of a 7 × 7 initial convolutional layer and a 3 × 3 max pooling layer, which are used for preliminary feature extraction of the input image. The residual block increases or reduces dimensionality using 1×1 convolution and employs shortcut connections to directly transfer low-level features to higher-level ones. The output convolutional layer can extract high-level semantic features. Finally, an embedding feature vector is generated through an average pooling layer. Multi-view features are assigned different weight values based on their importance, and a weighted average is calculated to aggregate these features.

ResNet is primarily designed for processing 2D image tasks. To adapt ResNet for 3D point cloud data, we project the 3D point cloud into 2D images from multiple viewpoints, denoted as follows: $\{S_{i}{\}}_{i=1}^{M}$ , where M represents the number of views. These projected images are resized 224×224×3 and then passed through the ResNet-50 network [14] to encode them into multi-view feature representations. After global average pooling, 1024-dimensional features are extracted for each view, and considering the varying importance of different views, as shown in Fig 3, we assign weight values to features from different views based on their importance. These weighted features are then averaged to obtain 2D multi-view features.

$$y_{i}=F(x_{i},\{W\})+x_{i},i=1,2...M$$ (6)

$$f_{2d}=\sum_{i=1}^{M}\alpha_{i}*f_{i}$$ (7)

As shown in Eq 6, the input *x* is directly added to *F*(*x*) through a shortcut connection, resulting in the output feature *y*. Here, *F*(*x*) represents the residual function computed by the residual block, which consists of convolutional layers and corresponding ReLU activations, with *W* denoting the weight parameters. By utilizing residual blocks, ResNet can construct very deep networks capable of learning complex and deep semantic features. Eq 7 demonstrates that averaging features from different views effectively fuses diverse types of information, resulting in more comprehensive feature representations. *α* represents the important weight values of different views.

Inspired by PointCLIP [12], which employs a small set of annotated data for fine-tuning to dynamically learn the importance of different views. Similarly, we observe significant variations in view contributions. Therefore, we leverage the CLIP model to adaptively assign view weights. This weighting strategy ensures that the fused multi-view features accurately represent the significance of each view, thereby enhancing the overall feature representation. We leverage the CLIP model, which achieves cross-modal feature alignment through joint training of image and text encoders without requiring specific labels. Building on this capability, we extract image features from the CLIP model and generate category embeddings using text prompts [36]. Subsequently, we compute the similarity between the image features and category embeddings. These similarities are normalized to produce probability distributions, which are then utilized to determine the classification confidence for each projected depth map. For each combination of features, the image features are adjusted according to the current viewpoint weights, and a new accuracy score is computed. If the current accuracy surpasses the previous best accuracy, the viewpoint weights are updated and recorded. To demonstrate the proposed method, we present the pseudo-code in Algorithm 1.

In the 3D encoder branch, DGCNN [13] dynamically utilizes the K-nearest neighbor to construct the graph-based neighborhood relationships. As shown in Eq 8, *p*_*i*_ and *p*_*j*_ represent the current point and its neighboring point, respectively. The relative coordinate difference between the current and neighboring points, given by *p*_*j*_ − *p*_*i*_, explicitly encodes local spatial geometric relationships.. The features of the current point are combined with the aggregated relative position features from multiple neighbors and are fed into a multi-layer perceptron (MLP), where they are transformed into a higher-dimensional space [37]. As shown in Eq 9, the transformed vectors are subjected to max-pooling, resulting in a 1024-dimensional global feature vector while ensuring the permutation invariance of the point cloud. By stacking multiple EdgeConv layers, DGCNN extracts local features and global spatial geometric information from point clouds.

$$h(p_{i},p_{j})=MLP(p_{i}⊕(p_{j}-p_{i}))$$ (8)

$$f_{3d}=MAX(h(p_{i},p_{j}))$$ (9)

### Class-specific feature selection by cross-attention

The class-specific features identified through this process are more universal and exhibit strong stability across different domains, thereby improving the model’s robustness in cross-domain scenarios. In this paper, we propose to achieve class-specific feature extraction through the fusion of 2D and 3D features. Since 3D spatial features and 2D semantic features provide complementary information [38], we utilize an attention mechanism to effectively integrate these features.

The attention mechanism assigns weights to different parts of a long input sequence, enabling the model to focus on and utilize the most relevant and effective information [39]. Specifically, we insert an attention module to capture the context-dependent interactions between 2D and 3D features. This module is designed to uncover semantic relationships and global spatial dependencies, facilitating a deeper integration of the two feature modalities. For a given input feature vector *X*, the Query (Q), Key (K), and Value (V) matrices are computed as follows:

$$Q=XW_{Q},K=XW_{K},V=XW_{V}$$ (10)

where *W*_*Q*_, *W*_*K*_, and $W_{V}$ denote the trainable weight matrices for the Query, Key, and Value projections, respectively. These weights are optimized via end-to-end backpropagation during training. The attention weights are optimized end-to-end using standard gradient descent, with gradients computed through the chain rule of the loss function. During this process, Q, K,and V is multiplied by different learnable weight matrices, then the attention mechanism dynamically adjusts the weights, allowing the model to automatically focus on the most important features. The output vectors are subsequently computed as defined in Eq 11, determining the importance weights of elements in the input sequence.

$$Attention(Q,K,V)=Softmax(\frac{Q\cdot K^{T}}{\sqrt{d_{k}}})V$$ (11)

The attention scores are computed as scaled dot-products between query (Q) and the key (K), where $\sqrt{d_{k}}$ serving as a scaling factor to mitigate gradient instability caused by excessively large dot product values. These scores are then normalized using a Softmax activation function to produce the attention weights. The final output feature vectors are generated through matrix multiplication between the normalized attention weights and the value matrix (V), resulting in the attention operation output *Attention*(*Q*,*K*,*V*). This process effectively performs a weighted summation of the value vectors to construct context-aware representations.

Cross-attention refers to the type of attention mechanism where the Query (Q) and Key/Value (K,V) come from different feature sets. The 2D feature will serve as inputs for *Q*_2*d*_, *K*_2*d*_, and $V_{2d}$; the 3D feature will serve as inputs for *Q*_3*d*_, *K*_3*d*_, and $V_{3d}$. In Eq 12, we first compute the dot product between the 2D query features *Q*_2*d*_ and the 2D key features *K*_2*d*_, then apply softmax normalization to obtain attention weights. These weights are subsequently multiplied by the 2D value features $V_{2d}$ to generate the output feature representation *f*_2*d*_. Eq 13 computes the cross-modal output vector by first calculating the dot product between *Q*_2*d*_ and *K*_3*d*_ and then applying softmax normalization to the resulting attention scores and finally adding this weighted attention pattern to the 3D value features $V_{3d}$ to generate the fused feature representation *f*_*cross*1_. Similarly, Eq 14 represents the interactive feature representation between 3D features. Eq 15 describes the cross-modal interaction between 3D and 2D features.

$$f_{2d}=Attention(Q_{2d},K_{2d},V_{2d})$$ (12)

$$f_{cross1}=Attention(Q_{2d},K_{3d},V_{3d})$$ (13)

$$f_{3d}=Attention(Q_{3d},K_{3d},V_{3d})$$ (14)

$$f_{cross2}=Attention(Q_{3d},K_{2d},V_{2d})$$ (15)

$$f=f_{2d}+f_{3d}+f_{cross1}+f_{cross2}$$ (16)

**Fig 4:**
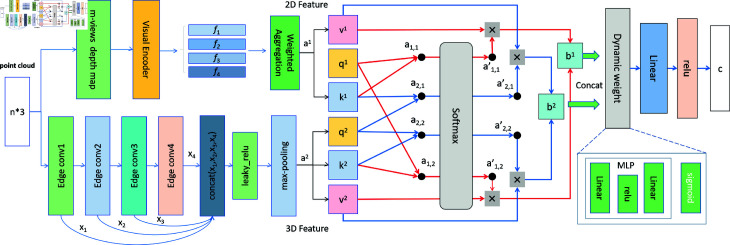
After feature extraction using ResNet in the 2D branch for multi-view features and DGCNN in the 3D branch for both global and local features, these extracted features will serve as the initialization inputs for the QKV in their respective 2D and 3D branches. The ${\text{a}}^{1}$ and ${\text{a}}^{2}$ represent 2D and 3D features, respectively. The attention mechanism is designed to identify the relevant connections between ${\text{a}}^{1}$ and ${\text{a}}^{2}$. First, multiplying *a* by different weight coefficients produces *q*, *k*, and *v*. Taking the dot-product of *q* and *k* yields an attention score representing the correlation between the two vectors. This score is normalized using softmax and scaled by multiplying with *v*. We calculate attention scores *a*_1,1_ and *a*_1,2_ using *q*_1_ with *k*_1_ and *k*_2_, respectively, and then multiply these scores by $v_{1}$ and $v_{2}$ accordingly. By summing these two results, we obtain ${\text{b}}^{1}$; similarly, we compute ${\text{b}}^{2}$. The resulting outputs, ${\text{b}}^{1}$ and ${\text{b}}^{2}$, represent the correlation between the two feature vectors. The output of the attention mechanism undergoes dynamic weight adjustment, allowing the model to adaptively focus on the most relevant features. After this adjustment, the output is passed through a linear layer to project it into a suitable representation space. The subsequent application of the ReLU activation function introduces non-linearity, enhancing the model’s ability to capture complex patterns and relationships within the data.

In Eq 16. we add four vectors, *f*_2*d*_, *f*_3*d*_, *f*_*cross*1_, and *f*_*cross*1_ , by performing element-wise addition across corresponding dimensions. This operation ensures that the resulting vector retains a dimensionality of 1024 while incorporating cross attention fusion information of 3D and 2D features. As illustrated in Fig 4, the detailed attention mechanism between the 2D and 3D vectors is presented. Specifically, the output *b*_1_ represents the correlation obtained by using 2D features to query key information from the 3D features, while *b*_2_ represents the correlation derived by using 3D features to query key information from the 2D features. The model can focus on the relevant high parts by different weight parameters to improve class-specific feature extraction selection.

## Experiments

### DataSet

We are validate our point cloud domain generalization results on the PointDA-10 datasets.

**Fig 5:**
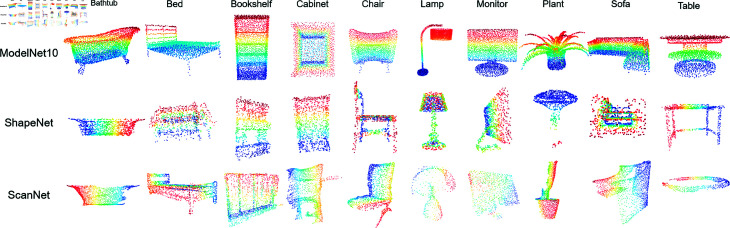
Comparison of Ten Classification Visualizations across Three Subsets (ModelNet10, ShapeNet, and ScanNet) of the PointDA-10 Dataset.

PointDA-10 is a point cloud dataset consisting of ModelNet-10 [40] (M), ShapeNet [41] (S), and ScanNet [42] (S ). The visualization comparison of the three subsets across 10 classification is presented in Fig 5. These three datasets contain the same ten categories (Bathtub, Bed, Bookshelf, Cabinet, Chair, Lamp, Monitor, Plant, Sofa, and Table), but the number of samples in each category is uneven. ModelNet10 and ShapeNet are sampled from 3D CAD point cloud object surfaces. ModelNet-10 appears denser as it represents geometric 3D shapes as a probability distribution of binary variables on a 3D voxel grid [40]. ScanNet is a richly-annotated dataset scanned from distinct real-world indoor scenes by RGB-D sensors [1]. The ScanNet dataset samples are obtained from real environments, which results in them being partially missing due to occlusion. Specially, the number of samples in the ShapeNet dataset is significantly greater than that in the other two datasets.

### Implementation details

Our framework utilizes DGCNN as the 3D feature embedding network for feature extraction and ResNet-50 as the 2D feature embedding network. We divide the source domain dataset into a training set and a validation set, allocating 80% and 20%, respectively, following the experimental protocol of DG [8]. All training is implemented on the source domain. We tune learning weight parameters by cross-validation in the source domain. The Adam optimizer [43] is used in Pytorch, we conducted a grid search across learning rates from 0.0001 to 0.001 and weight decay values from 0.00005 to 0.0005. The batch size is set to 32. We train the classification network for 150 epochs on a single NVIDIA RTX 3090 GPU. We conducted each experiment three times with random seeds and presented the average results. Our validation experiment includes two tasks: multi-source and single-source domains.

### Comparative methods

We compares our proposed method with other competing 3D DG methods in multi-source tasks, including DGCNN(w/o) [13], PointDAN [44], DefRec [45], GAST [46], and LCD [1]. Among these methods, PointDAN, DefRec, and GAST are original DA methods. For better comparison, we transfer these domain adaptation methods to the DG problem. The specific method is as follows: since the target domain is inaccessible in domain generalization problems, we omitted the alignment process between the source domain and the target domain. Simultaneously, we integrated the two source domains of these methods during training and directly evaluated the classification accuracy on the target domain. In the DGCNN(w/o) method, graph neural networks are utilized to directly extract point cloud features from the source domain and apply them to the target domain without any domain generalization process. This approach serves as a baseline for evaluation [1]. LCD [1] is an innovative multi-source DG method that leverages deformation reconstruction to augment the dataset while simultaneously extracting domain-invariant features from multiple sources. It represents a state-of-the-art approach for multi-source domain generalization. We compare our method with competing DG methods: MetaSets [8], PDG [47], SUG [21], GCM [48], and Push and Pull [6], which represent the advanced techniques in point cloud domain generalization research in recent years.

### Main results

We compare our method with other competing DG methods, as shown in Table 1, Our method outperforms all other methods in both average and individual results. Specifically, our method improves average accuracy by 6.1% compared to the baseline method DGCNN (w/o) and surpasses the state-of-the-art 3D DG method LCD [1] by 1.2%. It demonstrated the effectiveness of fusing 3D and 2D features to identify more discriminative class-specific features for domain generalization. Learning discriminative features from multiple source domains helps ensure the model’s decision boundary remains robust across different domains.

**Table 1:** Comparison of classification accuracy results(%) in multi-source task on PointDA-10.

Method	M, S →S	M, S→S	S, S →M	Avg.
MDAN [49]	74.5	45.2	57.1	58.9
DANN [50]	74.5	51.0	66.6	64.0
PointDAN [44]	79.2	39.5	79.6	66.1
DGCNN(w/o) [13]	80.5	47.3	83.5	70.4
GAST [46]	80.7	50.1	82.4	71.1
DefRec [45]	82.3	52.6	84.9	73.3
LCD [1]	85.7	53.2	86.9	75.3
Ours	86.4	55.9	87.3	76.5

In the single-source task, Table 2 presents a comparison of results between our method and other competing DG methods. Our method consistently outperforms the competing DG methods, achieving the highest average classification accuracy of 72.3%—a 2.6% improvement over the state-of-the-art Push and Pull method [6]. This highlights the effectiveness of cross-attention in capturing transferable contextual information, further demonstrating its effectiveness in single-source tasks as well. The discriminative features help the model find a suitable decision boundary, enabling it to perform well on both source and target domain data. By learning these discriminative features, the model can effectively classify data from the target domain, thereby avoiding overfitting to the source domain.

**Table 2:** Comparison of classification accuracy results(%) in single-source task on PointDA-10.

Method	M→S	M→S	S→M	S→S	S →M	S →S	Avg.
MetaSets [8]	86.0	52.3	67.3	42.1	69.8	69.5	64.5
PDG [47]	85.6	57.9	73.1	50.0	70.3	66.3	67.2
SUG [21]	82.8	57.2	74.8	52.2	73.1	69.5	68.3
GCM [48]	83.2	54.9	75.1	53.6	73.3	74	69.0
Push and Pull [6]	85.4	55.3	78.5	47.0	75.3	76.3	69.7
Ours	85.4	57.1	82.9	54.0	76.5	78.1	72.3

Table 3 presents the class-wise classification accuracy for the transfer task from ModelNet to ScanNet. Since ScanNet is derived from a real-world dataset with occlusions and missing parts, training the model solely on a 3D neighborhood-based network results in low class-wise accuracy. The multi-view projection compensates for the limitations in semantic feature extraction, enhancing the ability to distinguish similar categories, such as Bed and Bookshelf. Additionally, we significantly improved the distinction between the Chair and Sofa. However, we observe that different categories reflect different phenomena. The accuracies for the Chair and Sofa classes are significantly improved, whereas the accuracy for the Cabinet class is almost zero. This result arises from the inherent similarity between the Cabinet and Bookshelf categories. Our method intentionally enhances the generalization capability of model M, which improves its alignment with the target domain S  but simultaneously amplifies the inter-class similarity between these visually analogous categories. Consequently, Cabinet predictions naturally converge toward the Bookshelf class due to their shared structural features. This observation reveals a potential limitation in our cross-domain generalization approach.

**Table 3:** Class-wise classification accuracy(%) on ModelNet to ScanNet (M→S ) on PointDA-10.

Method	Bathtub	Bed	Bookshelf	Cabinet	Chair	Lamp	Monitor	Plant	Sofa	Table	Avg
Supervised	88.9	88.6	47.8	88.0	96.6	90.9	93.7	57.1	92.7	91.1	83.5
DGCNN(w/o)	59.4	1.0	18.4	7.4	55.7	43.5	84.8	60.0	3.4	39.7	37.3
SUG [21]	76.9	2.0	25.0	2.0	81.5	57.6	89.7	88.2	0.4	85.0	50.8
Ours	57.7	34.1	38.4	0.0	68.1	75.6	59.1	76.0	41.1	74.4	52.4

Table 4 presents the comparison of various metrics including Precision, Recall, F1 Score, and Accuracy for the single-source tasks on the PointDA-10 dataset. our method outperforms the baseline approach (without adaptation) in all metrics, indicating the effectiveness of our method.

**Table 4:** Comparison of metric results(%) in single-source task on PointDA-10.

Method	Metrics	M→S	M→S	S→M	S→S	S →M	S →S	Avg.
DGCNN(w/o)	Precision	65.78	35.70	81.94	33.98	68.66	51.55	56.27
	Recall	75.65	42.96	82.90	39.41	66.21	52.77	59.98
	F1 Score	70.37	38.99	82.42	36.49	67.41	52.16	57.97
	Accuracy	84.79	51.34	81.31	47.43	68.57	69.38	67.14
Ours	Precision	65.64	42.42	82.65	49.75	72.07	52.70	60.87
	Recall	74.13	52.43	83.46	43.81	74.23	61.09	64.86
	F1 Score	69.62	46.89	83.05	46.59	73.13	56.58	62.64
	Accuracy	85.03	57.09	82.83	54.21	76.51	78.29	72.33

**Fig 6:**
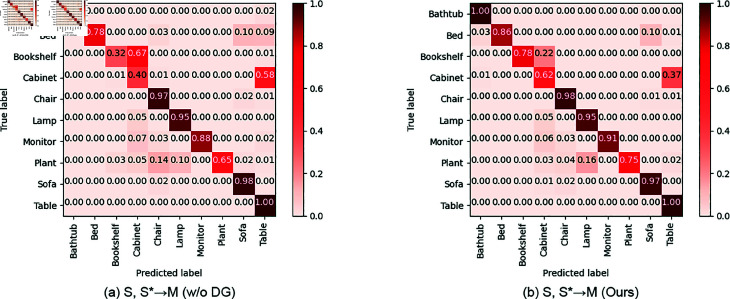
The comparison of confusion matrices for testing samples on the target domain for S, S →M is shown. The left matrix represents the baseline without DG, while the right matrix illustrates our method with DG.

Fig 6 presents the visualizations of the confusion matrices on the target domain, we evaluate the results of the S→S  task by comparing the baseline without DG to our method with DG. An examination of the confusion matrix for the baseline without DG reveals a relative domain shift. After applying our DG method, confusion is reduced for most classes, with the accuracy of the Bookshelf class improving significantly from 0.32 to 0.78. It reveals that learning discriminative features is beneficial for domain generalization.

**Fig 7:**
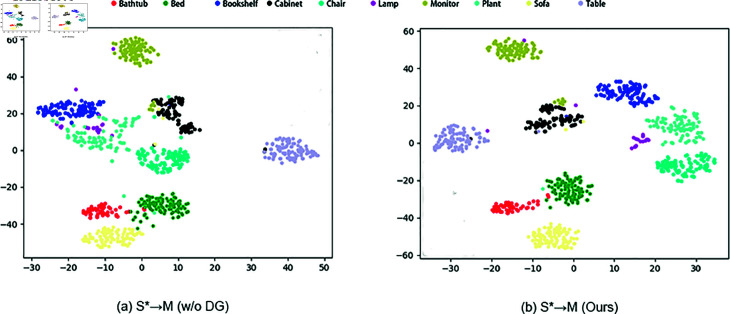
The t-SNE visualization of single source task DG, the model is trained in ScanNet and tested in ModelNet.

We visualize the feature distribution using t-SNE after training the model on ScanNet and testing it on ModelNet. As shown in Fig 7, the categories exhibit clearer distinctions, demonstrating that domain-invariant features have been successfully learned through cross-attention fusion, which also improves the model’s ability to learn generalized features in the single-source task.

### Quantitative analysis

#### Ablation study.

In this section, we perform a quantitative ablation study on PointDA-10 to assess the effectiveness of our proposed method. The evaluation focuses on two key aspects: the impact of the attention mechanism and the effectiveness of 2D-3D feature fusion. We designed two experimental schemes to explore feature fusion: one using a simple concatenation (concat) method to combine the feature sets, and the other leveraging an attention mechanism for feature fusion. To further evaluate the effectiveness of 2D and 3D feature fusion, we conducted extensive experiments comparing the fusion of 2D with 2D and 3D with 3D as baselines. Specifically, we tested the following configurations: (1) Concat (2D3D), (2) Att (3D3D), and (3) Att (2D3D). These ablation studies were conducted across both multi-source and single-source tasks to ensure comprehensive evaluation.

Table 5 presents the comparison of these variants in the multi-source task. The attention-based fusion method demonstrates higher effectiveness compared to the concatenation approach. Furthermore, Att (3D2D) outperforms the Att (3D3D), achieving the highest average accuracy among the tested methods, with an accuracy of 76.5%. These experimental results highlight the effectiveness of the attention-based fusion mechanism. By integrating 3D and 2D features, the attention mechanism identifies discriminative features in point clouds, enhancing the model’s ability to achieve robust generalization.

**Table 5:** Ablation study quantitative classification accuracy results(%) of our method in multi-sources task on PointDA-10.

Method	Con	Att	2D	3D	M, S →S	M, S→S	S, S →M	Avg.
DGCNN(w/o) [13]					80.5	47.3	83.5	70.4
PointDAN [44]					79.2	39.5	79.6	66.1
DefRec+PCM [45]					82.3	52.6	84.9	73.3
GAST [46]					80.7	50.1	82.4	71.1
LCD [1]					85.7	53.2	86.9	75.3
Ours+Concat(3D2D)	✓		✓	✓	82.8	53.4	82.0	72.7
Ours+Att(3D3D)		✓		✓	84.3	54.0	85.9	74.7
Ours+Att(3D2D)		✓	✓	✓	86.4	55.9	87.3	76.5

We also conducted experiments on a single-source domain, yielding consistent results. As shown in Table 6, the attention-based fusion of 2D and 3D feature achieves an average accuracy that outperforms the Concat(3D2D) and Att (3D3D) . This underscores the critical role of the attention mechanism in achieving robust domain generalization for both multi-source and single-source tasks.

**Table 6:** Ablation quantitative study of classification accuracy results(%) of our method in single-source task on PointDA-10.

Method	Con	Att	2D	3D	M→S	M→S	S→M	S→S	S →M	S →S	Avg.
PDG [47]					85.6	57.9	73.1	50.0	70.3	66.3	67.2
SUG [21]					82.8	57.2	74.8	52.2	73.1	69.5	68.3
GCM [48]					83.2	54.9	75.1	53.6	73.3	74.0	79.0
Push and Pull [6]					85.4	55.3	78.5	47.0	75.3	76.3	69.7
DGCNN [13]					84.7	49.1	80.0	47.3	72.8	76.7	68.4
DGCNN [13]+Def [45]					84.8	55.3	82.1	54.2	76.5	77.4	71.7
Ours+Concat(3D2D)	✓		✓	✓	83.9	54.7	81.9	53.9	75.7	77.5	71.3
Ours+Att(3D3D)		✓		✓	84.4	54.9	83.5	54.4	76.5	77.2	71.8
Ours+Att(3D2D)			✓	✓	85.0	57.1	82.9	54.0	76.8	78.1	72.3

To evaluate the impact of the number of 2D projection views on the selection of discriminative features, we conducted ablation experiments by varying the number of projection views in multi-source tasks. As shown in Table 7, we evaluate point cloud representations using 1, 4, 6, 8, 10, and 12 projection views to capture multi-view information comprehensively. The results demonstrate that classification accuracy on the target domain generally improves with an increasing number of views, with 10 views achieving optimal performance at 76.3% accuracy. Additionally, increasing the number of views leads to higher computational costs.

**Table 7:** Ablation studies (%) of projection view numbers on PointDA-10.

Numbers	M, S →S	M, S→S	S, S →M	Avg.
1	83.3	53.9	84.8	74.0
4	83.4	53.7	85.9	74.3
6	83.7	54.3	86.6	74.9
8	83.9	54.5	86.9	75.1
10	85.7	55.9	87.3	76.3
12	85.5	55.6	86.2	75.7

As presented in Table 8, we conducted a comprehensive comparison of the domain generalization performance between PointNet and DGCNN as backbone networks for point cloud data. The experimental results demonstrate that DGCNN achieves a classification accuracy of 72.3% on the target domain, which is significantly higher than the 67.1% accuracy attained by PointNet. This performance gap can be primarily attributed to DGCNN’s dynamic graph convolution mechanism, which excels in capturing both local geometric structures and global contextual relationships within point clouds, thereby enhancing the model’s generalization capability across diverse domains. In contrast, while PointNet effectively handles unordered point clouds through symmetric functions, its limited capacity to model intricate local features results in suboptimal performance in domain generalization scenarios. Based on these findings, we conclude that DGCNN offers superior advantages for point cloud domain generalization tasks.

**Table 8:** Comparison of classification accuracy results(%) in single-source task on PointDA-10.

Method	Backbone	M→S	M→S	S→M	S→S	S →M	S →S	Avg.
DGCNN(w/o)	PointNet	64.2	33.0	47.6	33.9	49.1	64.1	48.7
	DGCNN	83.3	43.8	75.5	42.5	63.8	64.2	62.2
PointDAN [44]	PointNet	83.9	44.8	63.3	45.7	43.6	56.4	56.3
GAST [46]	DGCNN	84.8	59.8	80.8	56.7	81.1	74.9	73.0
SLT [51]	DGCNN	86.2	58.6	81.4	56.9	81.5	74.4	73.2
PDG [47]	DGCNN	85.6	57.9	73.1	50.0	70.3	66.3	67.2
SUG [21]	PointNet	64.3	38.7	44.0	36.2	44.5	54.7	47.4
	DGCNN	82.8	57.2	74.8	52.2	73.1	69.5	68.3
Ours	PointNet	84.9	50.9	72.6	53.4	68.5	72.8	67.1
	DGCNN	85.4	57.1	82.9	54.0	76.5	78.1	72.3

#### Convergence study.

**Fig 8:**
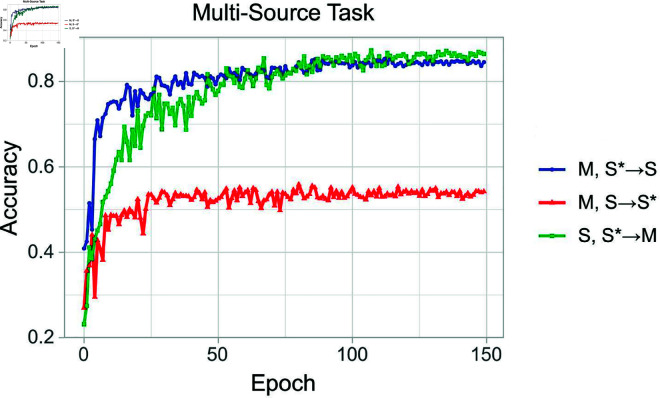
Convergence analysis: the experiment was conducted for 150 epochs on multi-source domain tasks, achieving convergence in all three sets of experiments.

We evaluated the convergence of the proposed method across three transfer tasks in the multi-source setting. As shown in Fig 8, convergence is achieved within 150 rounds. The attention fusion mechanism facilitates faster convergence and enhances stability during the process.

### Limitation

Our experimental results reveal a key trade-off between computational efficiency and model performance. As shown in Table 9, while our DG method requires 2.34× longer training time compared to the baseline (DGCNN [13]), this computational overhead is justified by its 4.9% improvement in accuracy. Importantly, the accuracy gain demonstrates our method’s enhanced ability to learn domain-invariant representations, which is crucial for real-world deployment scenarios where domain shifts are prevalent. For applications where model accuracy is prioritized over training efficiency this trade-off would be considered favorable. As shown in Table 10, our method incurs higher computational costs due to multi-view projection and feature fusion, leading to increased per-sample latency compared to the baseline. Nevertheless, it maintains real-time performance (< 50 ms/sample) while demonstrating significantly enhanced generalization capabilities across domains.

**Table 9:** Computational cost comparison(ms/sample) on PointDA-10.

Methods	M→S	M→S	S→M	S→S	S →M	S →S	Avg.
DGCNN(w/o)	6.52	6.70	7.27	6.96	7.19	6.32	6.83
Ours	15.49	16.25	16.56	15.98	16.53	15.48	16.05

**Table 10:** Real-time performance comparison (ms/sample) on PointDA-10.

Methods	M→S	M→S	S→M	S→S	S →M	S →S	Avg.
DGCNN(w/o)	11.98	11.37	2.81	3.91	8.16	11.16	8.23
Ours	33.86	33.88	8.23	11.90	23.35	33.91	24.19

## Conclusion

Domain Generalization is a challenging problem in the field of cross-domain learning. To better predict unseen target domains without any training on 3D point clouds, we designed a novel point cloud domain generalization method. We project the raw 3D data into multiple views, utilizing ResNet-50 to extract 2D geometric contextual information and DGCNN to capture spatial information from the point cloud. Additionally, we employ an attention mechanism to extract key information between 2D and 3D features. The experimental results demonstrated the effectiveness of our method, achieving new state-of-the-art performance in multi-source and single-source tasks. To further improve classification predictions for unseen domains, we plan to explore multi-modal fusion in future work by combining joint 2D multi-view features with text prompts to enhance the model’s generalization and robustness.
